# Effect of bimodal mesoporous carbon as PtRu catalyst support for direct methanol fuel cells†

**DOI:** 10.1039/d0ra05676f

**Published:** 2020-08-18

**Authors:** Gonzalo Montiel, Eduardo Fuentes-Quezada, Mariano M. Bruno, Horacio R. Corti, Federico A. Viva

**Affiliations:** Instituto Nacional de Tecnología Industrial Av. General Paz 5445 San Martín Buenos Aires Argentina; Departamento de Física de la Materia Condensada, Comisión Nacional de Energía Atómica Av. General Paz 1499, San Martín Buenos Aires Argentina viva@tandar.cnea.gov.ar; Instituto de Nanociencia y Nanotecnología, CNEA-CONICETCentro Atómico Constituyentes Av Gral Paz 1499, San Martin Buenos Aires Argentina; Instituto de Investigaciones en Tecnologías Energéticas y Materiales Avanzados (IITEMA), Universidad Nacional de Río Cuarto, Facultad de Cs. Exactas Físico Química y Naturales, Departamento de Química 5800 Río Cuarto Argentina mbruno@exa.unrc.edu.ar

## Abstract

Mesoporous carbons (MCs) with different pore sizes were synthesized and evaluated as a catalyst support for fuel cells. The MCs were obtained from resorcinol–formaldehyde precursors, polymerized in the presence of polydiallyldimethylammonium chloride (cationic polyelectrolyte) as a structuring agent and commercial silica (Sipernat® or Aerosil®) as the hard template. The MC obtained with Aerosil® shows a broad pore size distribution with a maximum at 21 nm. On the other hand, the MCs with Sipernat® show a bimodal pore size distribution, with a narrow peak centered at 5 nm and a broad peak with a maximum *ca.* 30 nm. All MCs present a high specific surface area (800–1000 m^2^ g^−1^) and total pore volume ranging from 1.36 to 1.69 cm^3^ g^−1^. PtRu nanoparticles were deposited onto the MC support by an impregnation–reduction method with NaBH_4_ at 80 °C in basic media. The electrochemical characterization reveals improved electrocatalysis towards the methanol oxidation for the catalyst deposited over the carbon with the highest total pore volume. This catalyst also presented the highest CO_2_ conversion efficiency, *ca.* 80%, for the methanol oxidation as determined by differential electrochemical mass spectroscopy analysis. Moreover, the catalyst as a fuel cell anode showed the best performance, reaching a power density of 125 mW cm^−2^ at 90 °C with methanol as fuel and dry O_2_.

## Abstract 2

A porous carbon with different pore sizes was synthesized and employed as support for PtRu nanoparticles.

## Graphical abstract

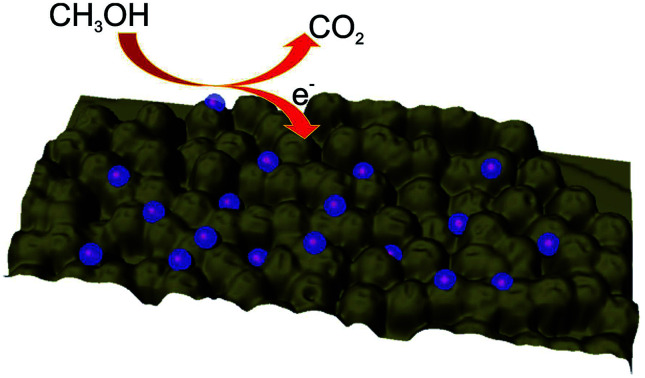

## 1 Introduction

Improving the performance of components that constitute a fuel cell (FC) is of great interest as it holds the promise of a convenient energy source for various types of applications.^1,2^ FCs are still ideal candidates to provide electrical energy with low to no environmental impact.^3^ Among the FCs operating at low temperature, the direct methanol fuel cell (DMFC) has attracted a great deal of attention, partly because of methanol's favorable properties such as high volumetric energy density and ease of handling as a liquid under ambient conditions.^1,2^ Moreover, this type of fuel cell are ideal candidate for portable applications.^4^ The core of a polymer electrolyte membrane fuel cell (PEMFC), is the membrane electrode assembly (MEA) that includes the catalytic layers (CL), gas diffusion layers (GDL) and the proton exchange membrane.^5,6^ The most commonly used catalysts in DMFCs are PtRu on the anode side^7–9^ and Pt on the cathode side, where the high cost of the noble metals is considered to be one of the main obstacles to commercialization.^10^

The metal catalysts in the CL are employed as nanoparticles dispersed over a conductive support. The most common support for fuel cell catalysts are carbon-based materials.^11–14^ The structure of the catalyst support can determine the catalyst nanoparticles stability and activity toward methanol oxidation.^15,16^ The support can maximize the particle dispersion as well as the electroactive catalyst area, while improves the mass transfer of reactants and products.^3,6^ Due to the mentioned cost of noble metals, the DMFC anode and cathode catalyst loadings must drop below 1.0 mg cm^−2^ from the present 2.0–4.0 mg cm^−2^ while maintaining the cell performance.^12^ Recently, different routes for the preparation of advanced nanostructured carbon materials have emerged, providing extra fine-tuning of the supported catalyst electroactivity.^12,13,17–19^

An ideal carbon support should allow the preparation of highly dispersed catalytic nanoparticles, whereas the porosity should ensure the ionomer penetration for proton transport while allowing a facile access path for reactants and by-products.^17,20^ It was shown that pores over 20 nm can ensure the formation of the triple phase boundary (TPB) region by allowing an optimal contact between the catalyst and the ionomer (Nafion).^21,22^ Additionally, the carbon surface defects, surficial groups and the microporosity have been identified as anchoring sites for metal nanoparticle, improving particle dispersion and catalytic activity.^23,24^ The porosity can also influence the residence time of the reactant and the by-products near of the catalyst nanoparticles.^12,25–27^ In this sense, a carbon material with hierarchical pore size distribution could offer a plausible way to guarantee the mentioned features. In previous works, we have demonstrated that carbon with micropores/mesopores obtained by using polyelectrolyte as a structuring agent can improve the catalyst performance in a fuel cell.^17,22,23,28^ More recently, we have presented a new method for the synthesis of carbon with dual mesopores size which can satisfy the requirements of an appropriate porous carbon support.^29^ This study analyzes the effect of the pore size distribution and the pore volume fraction of the carbon support on the fuel cell performance. The strategy was to produce carbons with a different pore size distribution and mesopore volume, preserving the rest of the textural properties. The set of properties were tuned by adding pore forming agents in the resorcinol formaldehyde polymerization media.^29–31^

In the present work, we describe the preparation and characterization of PtRu catalyst, synthesized by the impregnation–reduction method, supported on three different MCs. The carbon supports were obtained by carbonization of a resorcinol–formaldehyde polymer combining commercial silica as hard template and polydiallyldimethylammonium chloride as structuring agent. The MCs were characterized by N_2_ adsorption–desorption isotherms. The supported catalysts were characterized by powder X-ray diffraction (PXRD), transmission electron microscopy (TEM), and energy-dispersive X-ray spectroscopy (EDS). Stripping of CO was used for the determination of the catalyst electrochemical surface area (ECSA), whereas the electrocatalytic activity was determined by cyclic voltammetry (CV), chronoamperometry, and potentiodynamic differential electrochemical mass spectrometry (DEMS) measurements. Finally, the performance of the MEA with the prepared materials as anode catalyst were evaluated with methanol as fuel.

## 2 Experimental

### 2.1 Mesoporous carbon synthesis

Mesoporous carbons were obtained by carbonization of a resorcinol–formaldehyde (RF) resin. The polymer was prepared in the presence of sodium acetate as catalyst, a cationic polyelectrolyte as structuring agent (SA) and porous silica as hard template (HT). The SA employed was polydiallyldimethylammonium chloride (pDADMAC, 20% wt in H_2_O, average *M*_w_ = 100 000–200 000 g mol^−1^, Sigma-Aldrich), while the commercial silica powders Aerosil® 200 and Sipernat® 50 (EVONIK) were employed as the HT. The Aerosil® 200 is a nonporous fumed silica with a specific surface area between 50 and 500 m^2^ g^−1^, and a particles with sizes between 5 and 50 nm.^32^ The Sipernat® 50 are porous silica particles of 70 μm in diameter and a surface area of 475 m^2^ g^−1^.^29^ Briefly, two solutions were prepared, the solution A containing 2 g of resorcinol (*R*) (99.0% ACS, Sigma-Aldrich), 1 g of the SA solution, and 0.25 g of sodium acetate (trihydrate PA, Ciccarelli) were dissolved in 50 mL of milli-Q water. In the solution B, the HT was completely dispersed in 4 g of methanol (Biopack 99.8% wt), 5 g of glycerol (Biopack 99.5% wt), and 50 mL of milli-Q water. Both solutions were mixed and heated in a reflux system with magnetic stirring. The polymerization began by adding 1.4 g of a formaldehyde solution (F) (37% wt, Sigma-Aldrich) to the dispersion. After 45 min, another 3 g of F were added. The heating and magnetic stirring was maintained for 20 minutes and cooled it to 25 °C. Glycerol and methanol were added to the aqueous polymerization media as dispersant and wetting agents, respectively, rendering the polymer in a powder form. By this synthesis procedure, three different MC were obtained; MC A05 was synthesized by using 0.50 g of Aerosil® 200, while MC S15 and MC S30 were prepared by using 1.50 g and 3.00 g of Sipernat® 50, respectively. The composite resin was vacuum filtered from the solution, dried in a vacuum oven at 100 °C overnight and then carbonized under a N_2_ stream of 1 L min^−1^ in a tubular furnace (Indef model T-150) from 20 °C to 1000 °C at a heating rate of 3 °C min^−1^ and finally held at 1000 °C for 120 minutes. The HT from the carbon was removed by etching with 3 M NaOH solution at 60 °C for 24 h under constant stirring. MCs were washed with milli-Q water in Soxhlet apparatus until a neutral pH was obtained and finally dried in a vacuum oven at 110 °C overnight.

### 2.2 Supported catalyst preparation

The preparation of PtRu nanoparticles deposited over the MCs was carried out following a procedure previously described.^17,33^ The metal precursors solutions, H_2_PtCl_6_·6H_2_O (tetrahedron) and RuCl_3_·*X*H_2_O (Aldrich), were added to a slurry of the MCs while stirring for 30 min. The pH was adjusted to 8.0 with 1 M NaOH (PA, Merck) aqueous solution and heated to 80 °C. Once the temperature was reached, NaBH_4_ (granular 98%, Sigma-Aldrich) was added in a molar ratio of 5 : 1 (NaBH_4_ to metal salt) to the suspension. Heating was maintained for 2 h, followed by stirring for 24 h at room temperature. The powder obtained was washed with milli-Q water in a Soxhlet apparatus and finally dried in a vacuum oven at 80 °C for 24 h.

### 2.3 Mesoporous carbon and supported catalyst surface characterization

An ASAP 2020 (Micrometrics) instrument was used to measure the nitrogen adsorption/desorption isotherms at −196 °C. The Brunauer–Emmett–Teller (BET) equation was used for determining the specific surface area (*S*_BET_) while the volume of micropores (pores size <2 nm) was determined by applying the Dubinin–Radushkevich (DR) equation. The mesopore volume and pore size distribution (PSD) were obtained with the Barrett–Joyner–Halenda (BJH) method. The fractions of mesopore volumes were determined from the adsorption branch of the isotherm, whereas mesopore size distribution was calculated from the desorption branch. The total volume was calculated at a relative pressure *P*/*P*^0^ = 0.99.^34,35^

PXRD pattern of the catalysts were obtained using a Siemens D5000 diffractometer with a Cu Kα source operating at 40 kV and 30 mA. The angle extended from 20 to 100° with a step size of 0.02° and a counting time of 2 s. TEM images were acquired with JEOL 100 CX II, meanwhile EDS was performed using an SEM Philips 505 with EDAX detector to quantify the atomic ratio of Pt and Ru in the catalysts. Thermogravimetric analyses (TGA) were performed with a Q600 SDT Thermal Analyzer from TA Instruments controlled by Q Series software. The experiments were carried out using approximately 10 mg of sample in alumina pans under air atmosphere (Praxair A10.0XD-T 99.99%), with a heating rate of 10 °C min^−1^ and a gas flow rate of 50 mL min^−1^. The metal content on the supported catalysts were calculated from the difference between the initial and final weights.

### 2.4 Electrochemical measurements

**Fig. 1:**
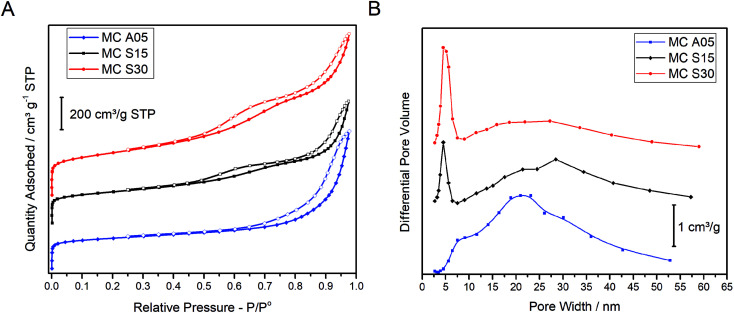
(A) N_2_ adsorption–desorption isotherms of the MCs measured at −196 °C. Closed symbols: adsorption branch, open symbols: desorption branch. (B) Pore size distribution obtained by applying the BJH method.

**Fig. 2:**
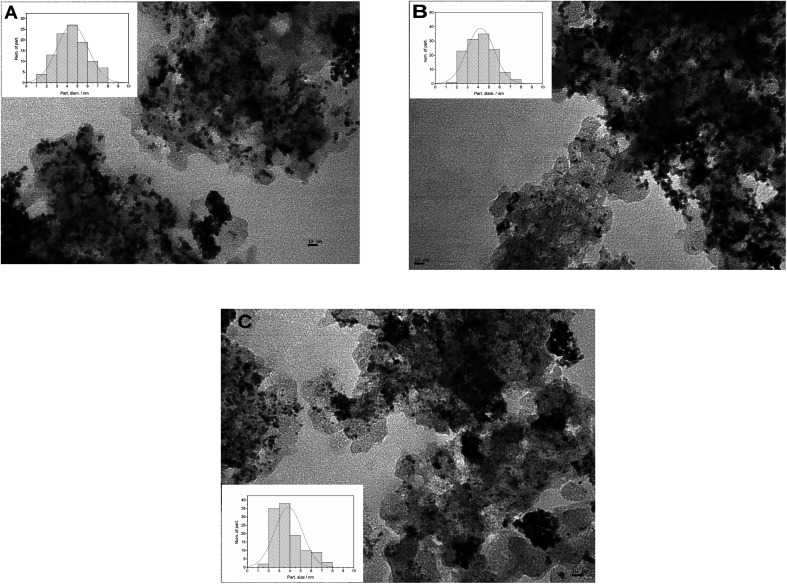
TEM images of the different supported catalysts (A) PtRu/MC A05, (B) PtRu/MC S15, (C) PtRu/MC S30. Inset: corresponding particle size distributions.

A suspension of the supported catalyst prepared in milli-Q water, isopropyl alcohol (Biopack) and Nafion ionomer (5% wt of Nafion dispersion in isopropyl alcohol, Aldrich) in a 0.15 : 1 ratio of Nafion to catalyst was deposited with a micropipette(1–10 μL, Rontaig) over the working electrode (WE), consisting of a glassy carbon disk (5 mm diam.) mounted on a Teflon rod. The WE was previously cleaned with isopropyl alcohol in an ultrasonic bath for 10 minutes and after deposition of the catalyst suspension, was dried in a vacuum oven at 80 °C for 10 minutes. CV, and chronoamperometry experiments were performed in a three-electrode cell. The counter electrode (CE) consisted of a coiled Pt wire 0.5 mm in diameter and 30 cm length, whereas a Ag/AgCl (sat. KCl) electrode was used as a reference electrode (RE). All potentials were converted against the standard hydrogen electrode (SHE). The upper potential limit for the voltammetric determinations was set to 0.8 V *vs.* SHE to avoid the formation of irreversible ruthenium oxides or Ru dissolution.^36^ The electrochemical surface area (ECSA) was measured by CO stripping voltammetry (ESI-Fig. 1†). The cell was filled with 0.5 M H_2_SO_4_ (95–97%, Merck) solution and saturated with CO (RG, Indura) for 45 min while the WE potential was maintained at 0.2 V *vs.* SHE. After the time elapsed, and while maintaining the potential, the solution was purged with N_2_ (RG, Indura) for 15 min to remove the unadsorbed CO, and immediately two scans between 0.05 and 0.8 V *vs.* SHE at a scan rate of 1 mV s^−1^ were performed. The catalysts ECSA was calculated based on the mass deposited onto the WE and the CV peak integral using the reference charge value of 420 μC cm^−2^ for the oxidation of a CO monolayer,^37^ and employed to convert the measured current (*i*) to current density (*j*). The catalysts CVs were performed on a 1 M methanol solution in 0.5 M H_2_SO_4_ by sweeping the potential between 0.05 and 0.8 V *vs.* SHE at 2, 5, 10 and 20 mV s^−1^ (ESI-Fig. 2†). The chronoamperometry were performed in the same electrolyte solution at 0.5 V *vs.* SHE for 1 h. These measures were employed to obtain the poisoning rate and the turn over frequency (TOF) as was calculated in previous reports.^38–40^ All electrochemical measurements were performed with an Autolab PGSTAT302N potentiostat.

#### 2.4.1 DEMS setup and cell configuration

The DEMS setup consisted of two differentially pumped chambers and a quadrupole mass spectrometer (100 amu, Pfeiffer). The primary vacuum chamber was pumped with a rotary vane pump (DUO 5, Pfeiffer) equipped with liquid N_2_ trap. The secondary chamber was pumped by a 60 L s^−1^ turbomolecular pump backed by a dry diaphragm pump (turbo drag pumping station TSH/U 071 E, Pfeiffer). A motorized gas-dosing valve (EVR 116, Pfeiffer) regulates the gas flow from the cell to the detector. The quadrupole mass spectrometer, equipped with a continuous dynode secondary electron multiplier/Faraday cup detector having a sensitivity of 200 A per mbar (QMS 200 M1, Prisma), was connected to the analysis chamber (secondary chamber).

The experiments were performed in a flow electrochemical cell designed for the DEMS.^17,41^ The WE consisted of a glassy carbon disk built ad hoc (6 mm in diameter) with a 1 mm diameter hole in the center through which the reactant flowed during the measurement. A suspension of the supported catalyst as described in Section 2.4 was spread over the working electrode (WE). The inlet port, from the cell to the DEMS pressure chamber, is through a stainless steel frit. The electrochemical cell opening is separated from the frit by a porous Teflon membrane (0.02 μm pore, 50 μm thickness, 50% porosity by Gore). The WE lies on top of the Teflon® membrane, separated from it by a 100 μm Teflon® gasket, allowing the formation of a thin liquid reacting layer. Volatile product species diffuse through the membrane to reach the mass spectrometer. The electrolyte flow rate (0.12 mL min^−1^) was controlled by a syringe pump (PC11U, Apema). Calibration by CO stripping and further quantification of the conversion efficiency was carried out as previously reported.^17,42^

### 2.5 MEA preparation and fuel cell testing

The synthesized PtRu/MCs were used as anode catalyst for the preparation of MEAs, while Pt supported over Vulcan carbon (Pt/VC) 60% (Fuel Cell Store) was used as cathode catalyst. A MEA with commercial PtRu catalyst over Vulcan carbon (PtRu/VC) 60% (Fuel Cell Store) as anode catalyst was also assembled for comparison. The catalyst suspension was prepared by mixing the catalyst with milli-Q water and Nafion ionomer solution (5% wt low MW alcohols, Ion Power) in a 1 : 10 : 7 mass proportion, respectively, and spread on one side of a 5 cm^2^ Toray C paper TGP-H 60 10% PTFE coated (Fuel Cell Technologies), for a final electrode loading *ca.* 3 mg cm^−2^. A Nafion 212 membrane (Ion Power) was placed in between the electrodes and hot pressed at 150 °C and 40 bar for 25 min. The Nafion membrane was previously treated by boiling in H_2_O_2_ 3% wt (H_2_O_2_ 30% wt, Bio-pack) followed by H_2_SO_4_ 3% wt (95–97%wt, Merck). The MEAs were mounted in a standard single cell housing with serpentine flow fields (Fuel Cell Technologies, Inc.). Teflon gasket films (50–150 μm) were employed as seal and the cell uniformly bolted with a torque of 2.3 Nm. After assembly of the cell, the MEA was re-humidified by circulating water at 80 °C overnight. Galvanodynamic polarization test was performed with a test station (University Test Station model from Fuel Cell Technologies) at 90 °C, from the open circuit voltage (OCV) to a voltage close of short circuit (0.05 V) while circulating 1 M methanol (Merk, HPLC grade) through the anode and dry O_2_ (RG 4.8, Indura) through the cathode. A Gilson Minipuls 3 peristaltic pump was used to circulate the methanol solution and a digital mass flow meter (MC 200 from Alicat Scientific) to control the O_2_ flows. For all the measurements the methanol flow was set to 2.0 mL min^−1^ while the O_2_ flow was 200 SCCM.

## 3 Results and discussion

### 3.1 Mesoporous carbons characterization

The textural properties of the MCs were analyzed by the nitrogen adsorption–desorption isotherms and the PSD as shown in Fig. 1(A and B), respectively. The isotherms of the three carbons show a similar profile at low relative pressure. The sharp rise at low *P*/*P*^o^ is indicative of the micropore presence. At higher *P*/*P*^o^, MC A05 shows a hysteresis loop in the adsorption–desorption isotherm, while two loops can be appreciated for MC S15 and MC S30. The single hysteresis loop for MC A05 and the double hysteresis loop for MC S15 and MC S30 indicates the monomodal and bimodal population of the mesopore size distribution, respectively. The MC S30 shows the highest adsorption volume. Fig. 1B shows the PSD for the three MCs. The plot for MC A05 presents a broad monomodal distribution with a maximum centered on 21 nm, whereas MC S15 and MC S30 show a narrow peak centered at 5 nm and a broad peak with a maximum *ca.* 30 nm. The textural properties obtained from the isotherms are summarized in Table 1. Based in the plots observed in Fig. 1B, two different mesopores sizes were arbitrarily defined: the small mesopores with pore diameter (d) between 2 and 7 nm and the large mesopores with *d* between 7 and 50 nm. All the MCs show high values of *S*_BET_, around 800 m^2^ g^−1^ for MC A05 and MC S15, and slightly higher for the MC S30 (1000 m^2^ g^−1^) due to the larger fraction of small mesopores and micropores. The micropore volume (*V*_m_) and the total pore volume (*V*_total_) are in the same order of magnitude for the three MCs. However, the main structural difference between the supports resides in the mesopore volumes. The volume of large size mesopores (*V*_l_), is higher for MC A05 followed by MC S30 and MC S15. Contrarily, the volume of the small mesopores (*V*_s_) is higher for MC S30 followed by MC S15 and MC A05.

**Table 1:** BET Surface area, volume of micro and mesopores, and total pore volume of the MCs

Sample	HT	HT/R	*S*_BET_ a (m^2^ g^−1^)	*V*_m_ b (cm^3^ g^−1^)	*V*_s_ c (cm^3^ g^−1^)	*V*_l_ d (cm^3^ g^−1^)	*V*_total_ e (cm^3^ g^−1^)
MC A05	Aerosil	0.25	796	0.33	0.11	0.77	1.50
MC S15	Sipernat	0.37	787	0.32	0.34	0.54	1.36
MC S30	Sipernat	0.75	1000	0.40	0.59	0.66	1.69

a Specific surface area using the BET method.

b Micropore volume from DR equation.

c Volume of small mesopores (2 < *d* < 7 nm).

d Volume of large mesopores (7 < *d* < 50 nm).

e Total pore volume at *P*/*P*° = 0.99.

The aforementioned results suggest that the specific synthesis method yield samples that present similar textural properties despite the different mesopore distribution, monomodal for the MC A05 and bimodal for the MC S15. Additionally, bimodal samples can be prepared with different ratios of small and large mesopores.

### 3.2 Catalyst surface characterization

The size and distribution of metal nanoparticles deposited on the different carbon supports were analyzed by TEM images, as shown in Fig. 2(A, B and C). Low-magnification TEM images show a good dispersion of catalyst particles for the three MCs supports. The catalyst particle size diameter distribution was obtained by measuring the diameter of 100 randomly selected particles with the software ImageJ. The Fig. 2 insets show the bar chart with the overlapping Gaussian distribution. The mean particle size obtained from the images were 4.5 nm for PtRu/MC A05, 4.2 nm for PtRu/MC S15 and 3.9 nm for PtRu/MC S30. As mentioned above, supports with small mesopores and/or micropores can serve as anchoring sites, decreasing the particle size and improving their dispersion. The PtRu nanoparticle size decreases as the small mesopore volume increases (Table 1). While the difference in the mean particle size is not as significant as the difference in *V*_s_, the particle size distribution observed in the bar charts (Fig. 2 Insets) is more representative of the tendency observed for *V*_s_.

**Fig. 3:**
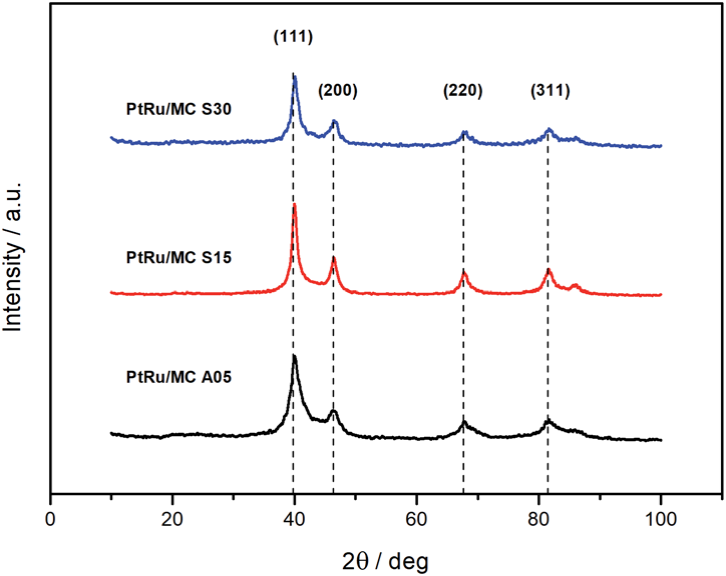
XRD diffractograms of the PtRu catalysts supported on MC A05, MC S15 and MC S30.

The PXRD diffractograms of the synthesized catalyst are shown in Fig. 3. The diffraction peaks at 2*θ* angles *ca.* 40, 46, 67, and 81 are due to face-centered cubic (fcc) crystalline Pt, assigned to the planes (111), (200), (220), and (311), respectively, with a slight shifting to higher 2*θ* values due to the presence of Ru.^43–45^ The lattice parameters of the metal nanoparticles were calculated by indexing the first three peaks yielding 3.900 ± 0.002 Å for PtRu/MC A05, 3.895 ± 0.002 Å for PtRu/MC S15, and 3.896 ± 0.002 Å for PtRu/MC S30. Using the reference value of 3.923 Å, the lattice parameter was used to estimate the Ru atomic fraction alloyed with Pt.^46^ The values obtained were 19% for PtRu/MC A05, 23% for PtRu/MC S15, and 22% for PtRu/MC S30, with an error of ±2%. The alloying Ru percentages, which are of the same order of magnitude for the three catalyst, shows that most of the Ru is present in an amorphous phase. Nonetheless, the percentage values are high for a catalyst that has not been subjected to a thermal treatment after deposition of the metal nanoparticles.^17,47^

The EDS results obtained for the catalyst indicated a Pt : Ru atomic ratio of 52 : 48 for PtRu/MC A05, 49 : 51 for PtRu MC S15, and 51 : 49 for PtRu/MC S30 close to the 1 : 1 atomic nominal ratio. The thermogravimetric analysis of the metal catalyst indicated metal loadings of 49% for PtRu/MC A05, 61% for PtRu MC S15, and 62% for PtRu/MC S30. The metal catalyst percentage over MC S15 and S30 are close to the intended value of 60% while is slightly lower over MC A05. As discussed above, the lower fraction of small size mesopores provides the least favorable support for particle anchoring.

### 3.3 Electrochemical catalyst characterizations

The ECSA values obtained from the CO striping voltammograms for PtRu/MC A05, PtRu/MC S15 and PtRu/MC S30 were 37 m^2^ g^−1^, 41 m^2^ g^−1^, and 42 m^2^ g^−1^, respectively. As in the case for the mean particle size, the difference between the three catalysts is small, nonetheless, the ECSA follows the expected trend were the lowest particle size correspond to the highest ECSA (PtRu/MC A05 < PtRu/MC S15 < PtRu/MC S30).

**Fig. 4:**
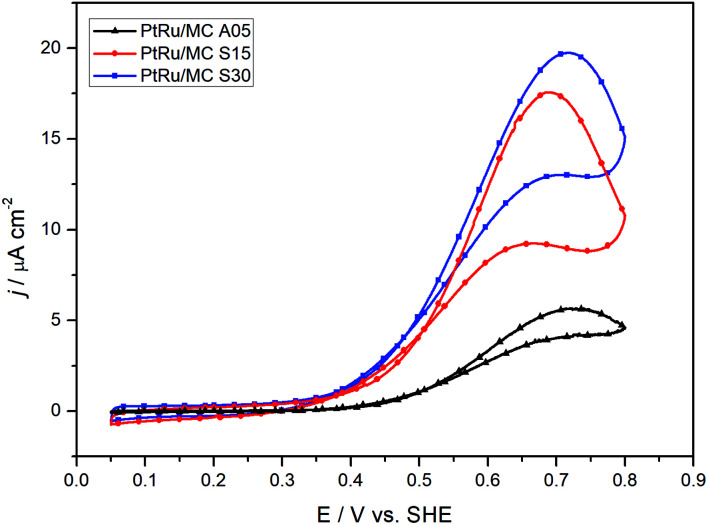
Cyclic voltammograms for the three different catalysts in 1 M methanol + 0.5 M H_2_SO_4_ at 2 mV s^−1^.

The catalytic activity of the prepared catalysts was assessed in a 1 M methanol + 0.5 M H_2_SO_4_ aqueous solution. Fig. 4 shows the voltammograms for the three catalysts at 2 mV s^−1^. The peak current density for the methanol oxidation increases in the order PtRu/MC S30 > PtRu/MC S15 > PtRu/MC A05, while the onset potential from the CVs are 0.49 V for the MC A05, 0.39 V for the MC S15, and 0.37 for the MC S30, following the same order. This suggest a more facile methanol oxidation on PtRu/MC S30.

**Fig. 5:**
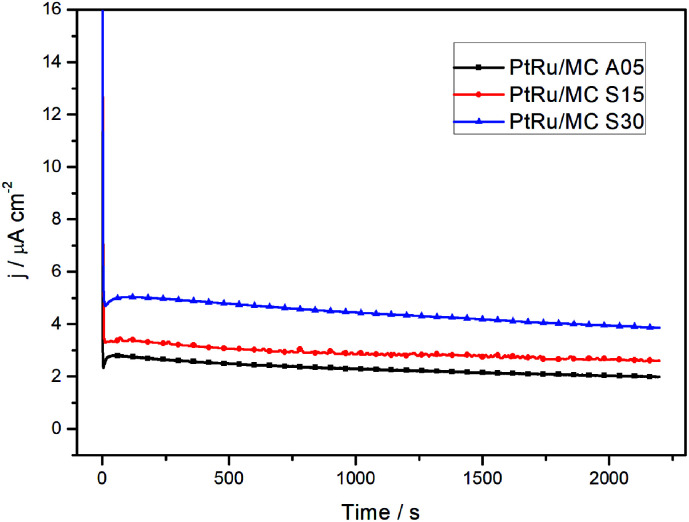
Chronoamperometry determination of the three different catalysts in 1 M methanol + 0.5 M H_2_SO_4_ at 0.5 V *vs.* SHE.

The chronoamperometry measurements of the catalysts at 0.5 V *vs.* SHE for a 30 min period are shown in Fig. 5. The rapid current decay shows a more restricted diffusion for PtRu/MC A05 than for PtRu/MC S30. The slope of the current transient in the chronoamperograms were employed for the determination of the catalyst poisoning rate (*δ*),^39,40^ while the steady-state current density was used for the calculation of the turnover frequency (TOF).^17,38,40^ The chronoamperometry slope between 500 and 1800 s has been attributed to the catalyst poisoning by CO, whereas at longer times the decay is related to anion adsorption.^17^ The values obtained for the poisoning rate and TOF, respectively, are presented in Table 2. The PtRu/MC S30 presents the highest number of reacting molecules per site, since TOF is directly proportional to the steady state current density.^40^ The TOF for the supported catalyst increases as the nanoparticle size decreases, *i.e.* as the support *V*_s_ increases. The results show the relationship between the turn over frequency and the nanoparticle size and therefore the relation with the support porosity. The poisoning rate shows similar results for the three catalysts. As the *δ* is related to the adsorption of intermediate species, the results would indicate that either the intermediates leave rapidly the catalyst surface, or the methanol oxidation produces a low amount of intermediates. The DEMS analysis, *vide infra*, shows that the catalyst conversion efficiency is high. Previous reported values of *δ* and TOF parameters suggest that mesoporous support allows the intermediates to rapidly leave the catalyst surface.^17,48–51^

**Table 2:** Poisoning rate (*δ*) and TOF of the three catalysts obtained from the chronoamperometry experiments

Catalyst sample	*δ* (% s^−1^)	TOF (molecules per site·per s)
PtRu/MC A05	0.0125	0.018
PtRu/MC S15	0.0083	0.023
PtRu/MC S30	0.0118	0.034

### 3.3.1 Catalyst characterization by DEMS

**Fig. 6:**
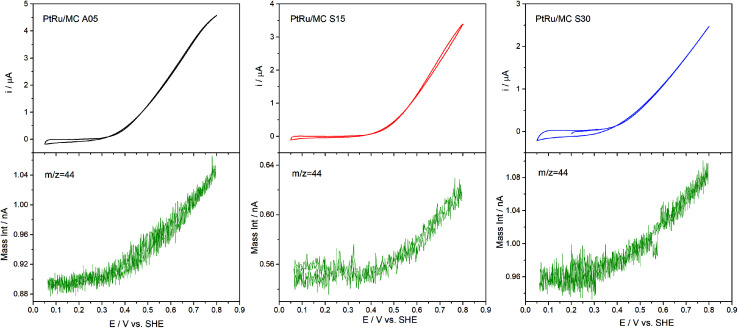
Potentiodynamic DEMS measurement of PtRu/MC A05, PtRu/MC S15 and PtRu/MC S30 in 1 M methanol + 0.5 M H_2_SO_4_ with the signal corresponding to *m*/*z* = 44 (CO_2_).

DEMS experiments were carried out to measure the conversion efficiency from methanol to CO_2_ for the synthesized catalysts. The electrochemical technique has been used to identify and quantify electrocatalysts reactions products for the methanol oxidation. The products directly identified by DEMS are CO_2_ (*m*/*z* = 44) (complete oxidation product) and methyl formate (*m*/*z* = 60), formed between the methanol present in the electrolyte and the formic acid generated during the electrochemical oxidation.^38,52,53^ Other oxidation products such as formaldehyde (*m*/*z* = 30), CO (*m*/*z* = 28) and formic acid (*m*/*z* = 46) exhibit signals that overlap with the fragments of methanol (*m*/*z* = 32) and CO_2_ present in the mass spectra, which complicates their direct determination.^38,52^Fig. 6 shows the results obtained, which include the electrochemical CVs and the mass signal *m*/*z* = 44 as a function of the applied potential. The electrochemical response acquired on the DEMS cell presented the expected shape for the CVs (Fig. 4) considering that the measures were carried out in a cell with a flowing thin layer of electrolyte.^17,42,54^ Moreover, the CVs shape in the DEMS cell allow to confirm that there is an adequate flow of electrolyte, avoiding any starvation of reactants during the measurements. From the mass signal (*m*/*z* = 44), the average current efficiency (*η*)^17^ for the formation of CO_2_ from methanol was calculated. The obtained values for the catalyst were 72% for PtRu/MC A05, 74% for PtRu/MC S15 and 84% for PtRu/MC S30. The results show that the catalysts supported over the bimodal carbons present a higher conversion of methanol to CO_2_ than the monomodal carbon. Moreover, for the PtRu/MC S30 catalyst, the signal for the *m*/*z* = 44 presents an onset at a lower potential (*ca.* 0.3 V) than the observed for the other two catalysts, indicating an improved conversion to CO_2_. As described in the previous sections, the three catalysts have a similar electrochemical surface area, while the main difference resides in the PSD and their contribution to the carbon total pore volume. The conversion efficiency results would indicate that the catalyst support affects the methanol oxidation, which can proceed *via* a parallel path mechanism: (1) *via* oxidation of adsorbed CO and (2) *via* dissolved intermediates.^8^ Under the flow conditions of the DEMS cell, any intermediate should be able to escape easily from the catalytic layer. However, it can be seen an increasing trend in catalyst performance and CO_2_ efficiency by increasing the mesopore content. The results are in agreement with the fact that methanol oxidation on PtRu proceeds *via* the direct pathway through adsorbed CO.^55,56^

### 3.4 Fuel cell performance characterizations

**Fig. 7:**
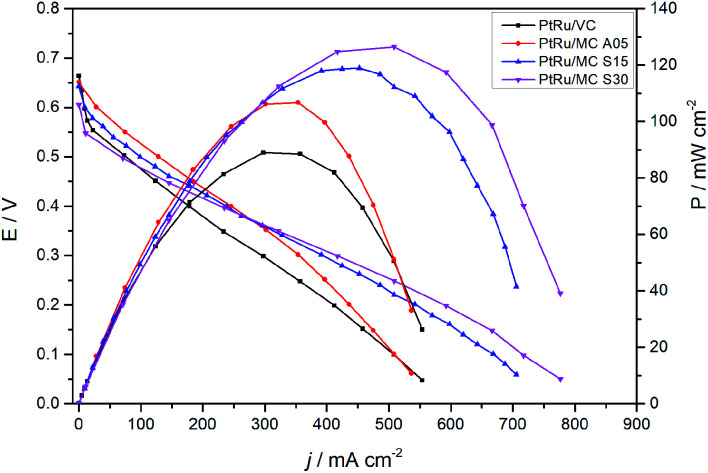
Polarization and power curves at 90 °C of the synthesized and commercial catalysts, measured with 1 M methanol (2.0 mL min^−1^ flow rate) at the anode and dry O_2_ (200 SCCM flow rate) at the cathode.

The fuel cell polarization and power curves are shown in Fig. 7. The plot presents the results of the MEAs prepared with the catalysts synthesized over the MCs and with a commercial catalyst for comparison. The cells with PtRu/MC A05 and PtRu/MC S15 as well as the one with PtRu/VC show an OCV of *ca.* 0.65 V while the one with PtRu/MC S30 presents a slightly lower OCV. The polarization curves show better performance for the cells with PtRu/MC S15 and PtRu/MC S30 as anode catalysts, *i.e.* those with catalyst over bimodal carbon, than the cell with PtRu/MC A05. The peak power density observed is 126 mW cm^−2^ for PtRu/MC S30, 115 mW cm^−2^ for PtRu/MC S15 and 109 mW cm^−2^ for PtRu/MC A05. For the commercial catalyst, the peak power density is 90 mW cm^−2^, which is an optimum value for the MEA composition and the experimental settings employed (temperature, O_2_ flow, methanol concentration) as compared with previously reported values.^12,13,20,57,58^

The polarization plots show a better performance of the catalyst deposited over the bimodal carbons compared to the monomodal support. The PtRu/MC S30, which has the highest amount of small mesopores (Fig. 1B and Table 1), shows the highest power density followed by PtRu/MC S15. The PtRu/MC A05 presents the lowest performance of the prepared catalysts in this report, but still it performs better than a previously reported PtRu catalyst deposited over a monomodal MC.^17,22^ The reported MC, synthesized by a process without hard template, exhibited a PSD with a narrow peak centered at 20 nm and a total pore volume of *ca* 1 cm^3^ g^−1^. The commercial catalyst, with Vulcan® carbon as support, displays the lowest performance. Vulcan® is a carbon black with a low surface area (252 m^2^ g^−1^) without mesoporosity or microporisity (*V*_T_ = 0.63 cm^3^ g^−1^).^14^ The measured fuel cell polarizations indicates that the support's mesoporosity, particularly the small mesopores, have a positive influence on the methanol oxidation, improving the overall cell performance.

## 4 Conclusions

In the present work, anode catalysts for DMFC formed by PtRu nanoparticles deposited on porous carbon with different pore size distribution were synthesized. A synthesis method employing silica as hard template and PDMAC as structuring agent was used to produce a hierarchical structure. This method produced MCs with similar textural properties (*S*_BET_, *V*_m_, *V*_T_) and different mesopore distributions. In the presence of PDMAC, Aerosil® produced a carbon with a single broad peak pore distribution centered at 21 nm, while with Sipernat® the PSD shows two peaks; a narrow one centered at 5 nm and a broad one with a maximum *ca.* 30 nm.

The electrochemical results and the fuel cell performance measurements show the effect of the support textural properties on the PtRu nanoparticles catalytic properties. The carbon support synthesized presents an adequate surface area and microporosity that provides good particle size and distribution across the surface. The presence of the mesopores also allows the formation of the TPB and a sponge like structure which facilitates the access of methanol onto the catalyst and the departure of CO_2_.

## Conflicts of interest

There are no conflicts of interest to declare.

## Supplementary Material

RA-010-D0RA05676F-s001
