# Supplementary material for: Effect of bimodal mesoporous carbon as PtRu catalyst support for direct methanol fuel cells
Source: RSC Adv. 2020 Aug 18;10(51):30631–9. doi: 10.1039/d0ra05676f (PMC9056354; doi:10.1039/d0ra05676f)
Supplement: RA-010-D0RA05676F-s001 [file RA-010-D0RA05676F-s001.pdf]

Electronic Supplementary Material (ESI) for RSC Advances.
This journal is © The Royal Society of Chemistry 2020

Effect of bimodal mesoporous carbon as PtRu catalyst support for direct methanol fuel cell

Gonzalo Montiel[1,2,3], Eduardo Fuentes-Quezada [2,3], Mariano M. Bruno[4], Horacio R. Corti[2,3], Federico A Viva[2,3]

1- Instituto Nacional de Tecnología Industrial, Av. General Paz 5445 San Martín Buenos Aires, Argentina.

2- Departamento de Física de la Materia Condensada, Comisión Nacional de Energía Atómica. Av. General Paz 1499, San Martín, Buenos Aires, Argentina.

3- Instituto de Nanociencia y Nanotecnología, CNEA-CONICET

Centro Atómico Constituyentes, Av Gral Paz 1499, San Martin, Buenos Aires, Argentina

4-Instituto de Investigaciones en Tecnologías Energéticas y Materiales Avanzados (IITEMA), Universidad Nacional de Río Cuarto, Facultad de Cs. Exactas Físico Química y Naturales, Departamento de Química, 5800 Río Cuarto, Argentina.

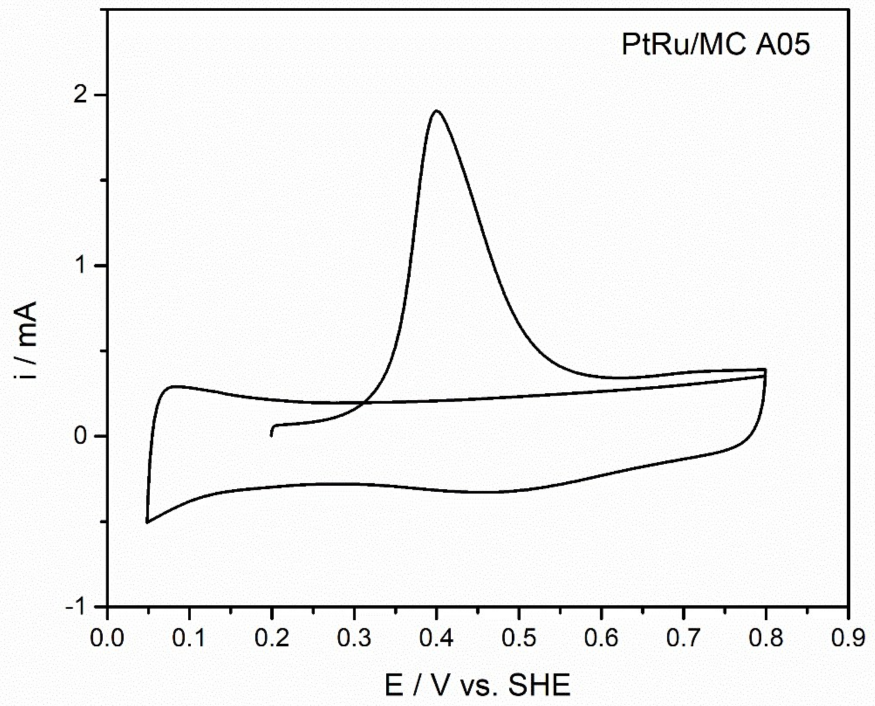
PtRu/MC A05
i / mA
2
1
0
-1
0.0 0.1 0.2 0.3 0.4 0.5 0.6 0.7 0.8 0.9
E / V vs. SHE

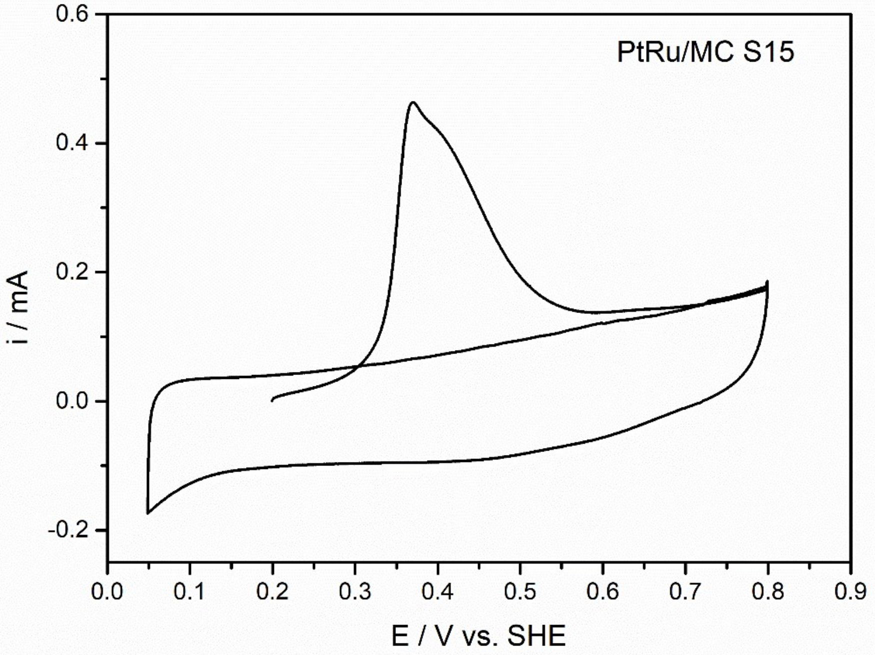
PtRu/MC S15
i / mA
0.6
0.4
0.2
0.0
-0.2
0.0 0.1 0.2 0.3 0.4 0.5 0.6 0.7 0.8 0.9
E / V vs. SHE

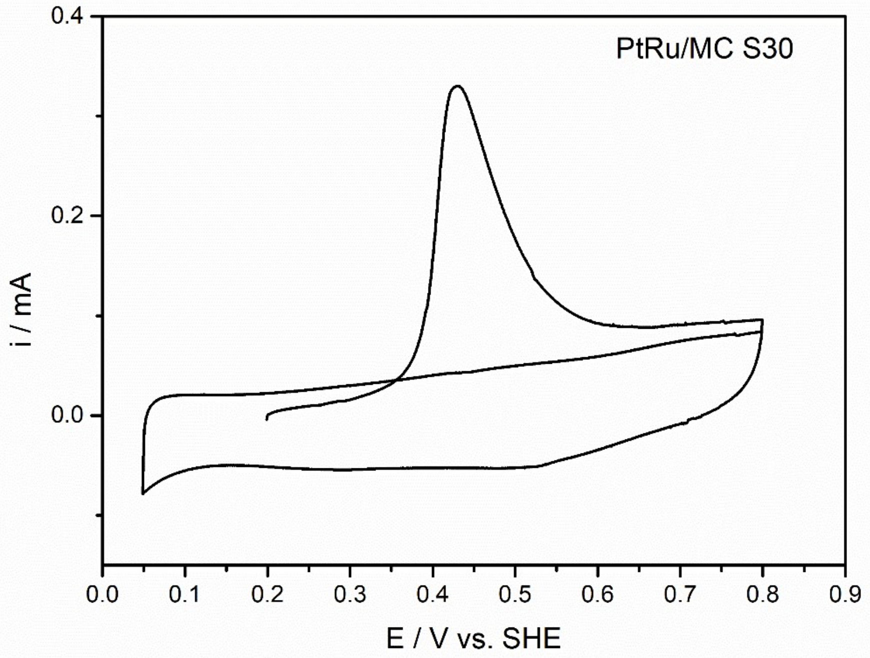

Figure S1. CO stripping voltammograms in 0.5 H_2SO_4 at 1 mV·s^{-1} for the synthesized catalyst

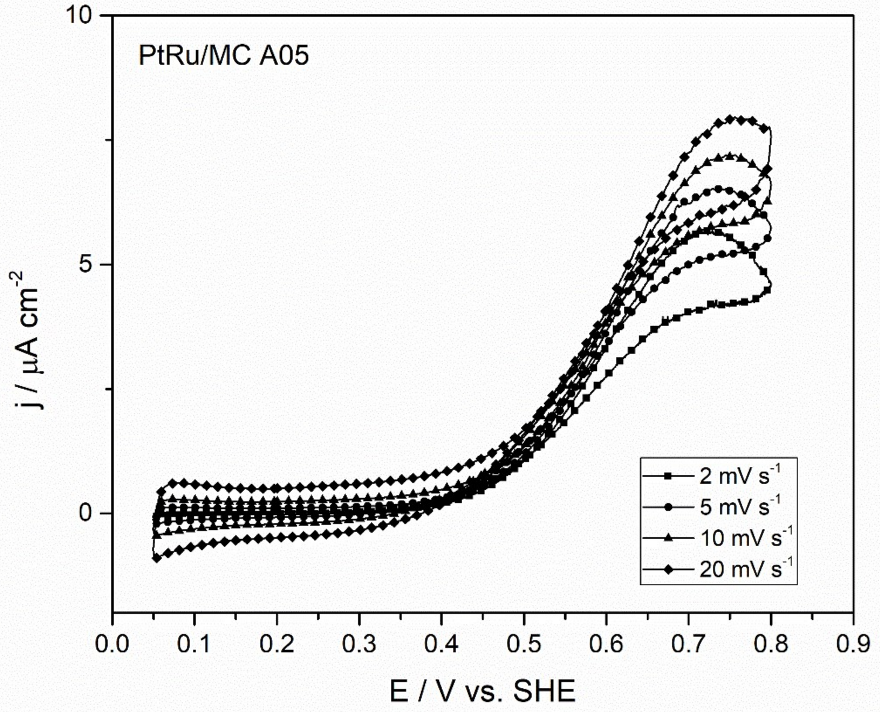
PtRu/MC A05
j / μA cm-2
E / V vs. SHE
2 mV s-1
5 mV s-1
10 mV s-1
20 mV s-1
10
5
0
0.0
0.1
0.2
0.3
0.4
0.5
0.6
0.7
0.8
0.9

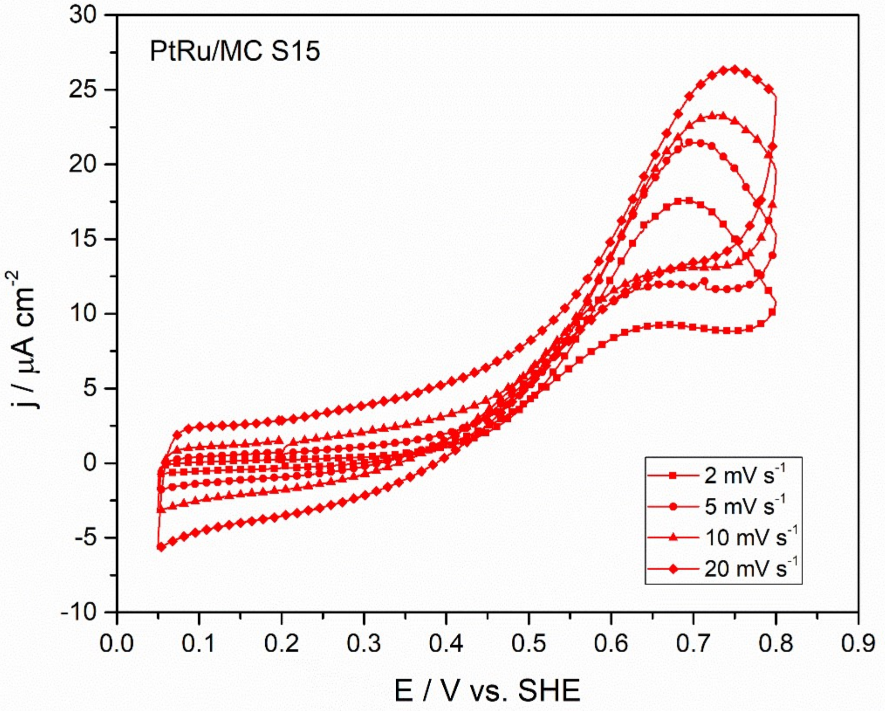
PtRu/MC S15
j / μA cm-2
E / V vs. SHE
2 mV s-1
5 mV s-1
10 mV s-1
20 mV s-1
30
25
20
15
10
5
0
-5
-10
0.0
0.1
0.2
0.3
0.4
0.5
0.6
0.7
0.8
0.9

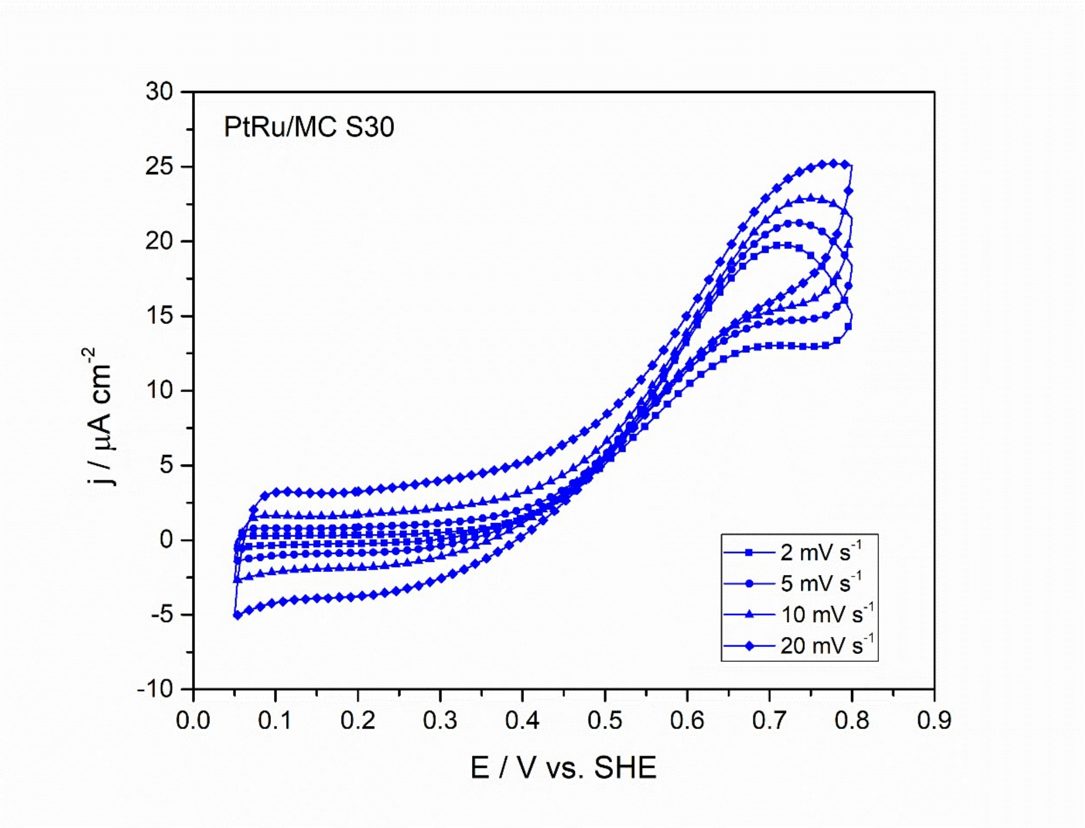

Figure S2. Cyclic voltammograms for the three different catalysts in 1 M methanol + 0.5 M H_2SO_4 at various voltage sweeping speeds.
